# Biological age predicts dementia and Alzheimer's disease plasma biomarker levels

**DOI:** 10.1002/alz70855_107627

**Published:** 2025-12-24

**Authors:** Jaclyn M. Eissman, Min N Qiao, Vrinda Kalia, Yiyi Ma, Marielba Zerlin‐Esteves, Dolly Reyes‐Dumeyer, Angel Piriz, Saurabh Dubey, Renu Nandakumar, Annie J. Lee, Raphael A Lantigua, Martin Medrano, Diones Rivera Mejia, Lawrence S. Honig, Clifton L. Dalgard, Gary W Miller, Richard Mayeux, Badri N. Vardarajan

**Affiliations:** ^1^ The Taub Institute for Research on Alzheimer's Disease and the Aging Brain, Columbia University, New York, NY, USA; ^2^ Gertrude H. Sergievsky Center, Columbia University, New York, NY, USA; ^3^ Mailman School of Public Health, Columbia University, New York, NY, USA; ^4^ Vagelos College of Physicians and Surgeons, Columbia University, New York, NY, USA; ^5^ School of Medicine, Pontificia Universidad Catolica Madre y Maestra, Santiago, Santiago, Dominican Republic; ^6^ CEDIMAT, Plaza de la Salud, Santo Domingo, Santo Domingo, Dominican Republic; ^7^ Universidad Pedro Henríquez Urena, Santo Domingo, Santo Domingo, Dominican Republic; ^8^ The American Genome Center, Center for Military Precision Health, Uniformed Services University of the Health Sciences, Bethesda, MD, USA; ^9^ Physiology & Genetics, Uniformed Services University of the Health Sciences, Bethesda, MD, USA

## Abstract

**Background:**

Advancing chronological age is a strong contributor to Alzheimer's disease (AD) risk, yet individual variability exists in aging trajectories. Novel AD biomarkers are needed to better stratify AD risk, especially in preclinical stages. To this end, we computed epigenetic “clocks,” a measure of biological age based on DNA methylation markers, and tested associations with dementia and AD biomarkers.

**Method:**

We leveraged whole blood and brain DNA methylation data collected from the EFIGA aging and AD cohort along with the Methylclock R package to compute Horvath and Hannum aging clocks (*N* = 731). Next, we calculated age acceleration, by residualizing the regression of biological age on chronological age. We ran linear regression models testing associations between biological age and age acceleration with a panel of plasma dementia and AD biomarkers. All regressions were adjusted for multiple comparisons (false‐discovery rate [FDR]).

**Result:**

Biological age was significantly correlated with chronological age (R^2^_Horvath_=0.18, p_Horvath_ =4.74x10^‐33^; R^2^_Hannum_=0.17, p_Hannum_=3.48x10^‐30^) and with sex (β_Horvath_=0.21, p_Horvath_=8.44x10^‐3^; β_Hannum_=0.27, p_Hannum_=7.32x10^‐4^). We replicated correlations in brain (R^2^_Horvath_=0.52, p_Horvath_=6.27x10^‐89^; R^2^_Hannum_=0.21, p_Hannum_=6.46x10^‐30^), and in an independent aging and AD cohort, ROS/MAP (in brain ‐ R^2^_Horvath_=0.49, p_Horvath_=6.51x10^‐78^; R^2^_Hannum_=0.34, p_Hannum_=1.03x10^‐47^). Seven plasma biomarkers were significantly associated with biological age calculated with the Horvath clock, the Hannum clock, or both: *p*‐tau217 (β_Horvath_=0.13, p.FDR_Horvath_=2.69x10^‐3^; β_Hannum_=0.19 p.FDR_Hannum_=1.35x10^‐5^), *p*‐tau181 (β_Horvath_=0.13, p.FDR_Horvath_=1.75x10^‐3^; β_Hannum_=0.14 p.FDR_Hannum_=1.75x10^‐3^), GFAP (β_Horvath_=0.09, p.FDR_Horvath_=2.07x10^‐2^; β_Hannum_=0.15 p.FDR_Hannum_=5.77x10^‐4^), *p*‐tau231 (β_Horvath_=0.11, p.FDR_Horvath_=9.87x10^‐3^), NfL (β_Hannum_=0.11, p.FDR_Hannum_=9.87x10^‐3^), NfL (β_Hannum_=0.11, p.FDR_Hannum_=9.87x10^‐3^), Aβ40 (β_Horvath_=0.09, p.FDR_Horvath_=2.07x10^‐2^; β_Hannum_=0.11, p.FDR_Hannum_=1.66x10^‐2^), and total‐tau (β_Horvath_=0.09, p.FDR_Horvath_=2.07x10^‐2^).

**Conclusion:**

We demonstrated that biological age is a predictor of AD pathology in preclinical and clinical stages, and we will next evaluate biological age acceleration as a predictor of pathology. In addition, we will be replicating all biomarker associations in brain by testing associations with autopsy measures of neuropathology. Future directions will include conducting models stratified by sex and by *APOE*‐ε4 carrier status to identify if the relationship between biological aging and AD biomarkers is modulated by biological sex or *APOE*‐ε4 genotype.

